# Bilayer or Not: Revisiting the Structural Nature of HBV Subviral Particles

**DOI:** 10.1002/jmv.70212

**Published:** 2025-02-05

**Authors:** Haitao Liu, Sonal Garg, Jianming Hu, Joseph Che‐Yen Wang

**Affiliations:** ^1^ Department of Microbiology and Immunology Penn State College of Medicine Hershey Pennsylvania USA; ^2^ State Key Laboratory of Pathogen and Biosecurity, Beijing Institute of Microbiology and Epidemiology, AMMS Beijing China

## Abstract

Hepatitis B virus (HBV) subviral particles (SVPs) are primarily stabilized by protein–protein interactions, with discrete lipid patches playing a supporting role, rather than forming a conventional lipid bilayer, challenging classical assumptions about viral membrane architecture.

Hepatitis B surface antigen (HBsAg) is a serological marker for HBV infection. It comprises three transmembrane proteins, S‐, M‐, and l‐HBsAg, which share identical C‐terminal regions but vary in the length of their N‐terminal extension. HBsAg can be incorporated into the virion envelope or assembles into SVPs with lipid derived from host cells [1]. The morphogenesis of SVPs produces two distinct forms: spherical and filamentous particles. Notably, in HBV carriers the abundance of SVPs is typically at 1000 to 10 000 times greater than infectious virions. Although the exact function of this surplus of SVPs is unclear, it has been postulated that the excess amount of SVPs may act as decoys, binding to host antibodies directed against HBsAg and thereby protecting infectious virions from neutralization and clearance by these antibodies [1].

In addition to their role in infection, HBV SVPs have important clinical applications. They serve as the foundation of licensed vaccines to prevent hepatitis B infection. When engineered with foreign antigenic epitopes, chimeric SVPs act as a platform to deliver and boost the immune response against medically relevant pathogens, as demonstrated in a vaccine against malaria [2]. Furthermore, SVP, in combination with HBsAg, exhibits pronounced immune‐inhibitory effects, modulating innate immunity [2], as well as B‐cell activity [3], and T‐cell functions [4, 5]. Recently, an antiviral strategy targeting the assembly and release of SVPs, or using HBsAg‐suppressive siRNA combined with interferon‐alpha, has been shown to achieve a significant functional cure rate of HBV infection [6, 7]. Despite these multifunctional roles, detailed structural information regarding the spatial arrangement of HBsAg within SVP was still missing until most recently [8, 9]. Emerging hypotheses propose spherical SVPs bud from the ER‐Golgi intermediate compartment [10]. In contrast, filamentous SVPs and virions are secreted through the endosomal sorting complex required for transport and multivesicular bodies [11]. While additional research is required to validate these mechanisms, the prevailing assumption is that the membrane organization in SVPs is similar to that of infectious virions.

Although HBV is classified as an enveloped DNA virus, its mechanism of membrane fusion during infection remains poorly understood, creating a substantial gap in our understanding of its molecular virology. HBV SVPs, which share the same envelope proteins and lipids as the virus, are key to addressing this knowledge gap [11, 12, 13]. Elucidating the structural organization of the SVP membrane is, therefore, a crucial step forward in clarifying its functional roles. However, earlier efforts to characterize SVP structures have been limited to low‐resolution studies (12–30 Å), which yielded inconsistent and contradictory interpretations. In 2022, Liu et al. used cryo‐EM to resolve the first subnanometer‐resolution structures of woodchuck hepatitis virus (WHV) SVPs from experimentally infected woodchucks and HBV SVPs from chronically infected patients [8]. Unlike the icosahedral symmetry of capsids, both WHV and HBV SVPs exhibit a rhombicuboctahedral geometry, approximately 23 nm in diameter, with 24 protruding spikes on their surface. Each spike is composed of asymmetric dimers of S‐HBsAg, and each subunit contains four helical domains. A total of 48 S‐HBsAg subunits assemble symmetrically into an SVP, forming a tightly packed particle stabilized by an extensive network of salt bridges, hydrogen bonds, and hydrophobic interactions.

The cryo‐EM density maps revealed two distinct layers: an outer layer with continuous density and an inner layer forming a complex protein network. These layers were determined to primarily consist of protein rather than lipids, consistent with the idea that the HBV virus is a structured assembly where lipids interact with hydrophobic protein regions to create a stable, protective framework [14]. Unlike canonical lipid bilayers, the outer layer incorporates discrete lipid patches interspersed among helical protein domains. These lipid moieties interact with hydrophobic surfaces on the S‐HBsAg dimers, contributing to particle stability. In contrast, the inner layer is protein‐dense and forms a tightly packed hydrophilic network. The SVPs are naturally arranged such that hydrophilic surfaces are exposed to the exterior and interior surfaces of the particle, while hydrophobic regions are buried within the structure, interacting with lipid patches. This spatial organization protects the hydrophobic areas from exposure to the aqueous environments and further stabilizes the particle. Even when treated with detergent to remove lipids, SVPs retain their structural integrity, with only partial unwound helices, indicating that the assembly is largely driven by protein‐protein interactions and is inherently stable unless exposed to denaturing agents [8]. These findings align with previous studies, which show that lipid extraction does not disrupt SVP morphology or antigenicity, highlighting that structural stability arises primarily from protein–protein interactions rather than a lipid bilayer framework [15]. Furthermore, complementary analyses using electron spin resonance and fluorescence spectroscopy support a patchy lipid distribution or monolayer arrangement, where lipids stabilize hydrophobic protein regions rather than forming a continuous bilayer [16].

Recently, Wang et al. published a research article “Inherent symmetry and flexibility in hepatitis B virus subviral particles” in *Science* [17]. In this study, the authors proposed that HBV SVPs possess a conventional lipid bilayer based on their cryo‐EM density interpretations. While the work provides valuable insights into SVP assembly and subunit flexibility, the evidence presented fails to support a classical bilayer structure. The conclusions on membrane organization were drawn from two approaches: (1) cryo‐EM density maps obtained during intermediate data processing, and (2) difference density calculations, where high‐resolution atomic models were subtracted from noisy cryo‐EM data. Despite resolving S‐HBsAg at high resolution, the resulting atomic models (PDB: 8YMJ, 8YMK) revealed highly hydrophilic protein surfaces, which are intrinsically incompatible with the hydrophobic environment required for a lipid bilayer (Figure 1). This fundamental discrepancy raises critical questions about how such protein structures could integrate into a conventional lipid bilayer framework. Notably, lipid densities are generally weaker than proteins in cryo‐EM due to their lack of ordered structural elements and higher fluidity. However, the cryo‐EM maps of HBV SVPs exhibit density intensities for inner and outer layers identical to the transmembrane and protruding protein regions, with characteristics inconsistent with a traditional lipid bilayer [8]. By contrast, small virus‐like particles from Dengue virus, which also utilize ER membranes, display a clear lipid bilayer with distinct density differences between lipids and proteins [18].

**Figure 1:**
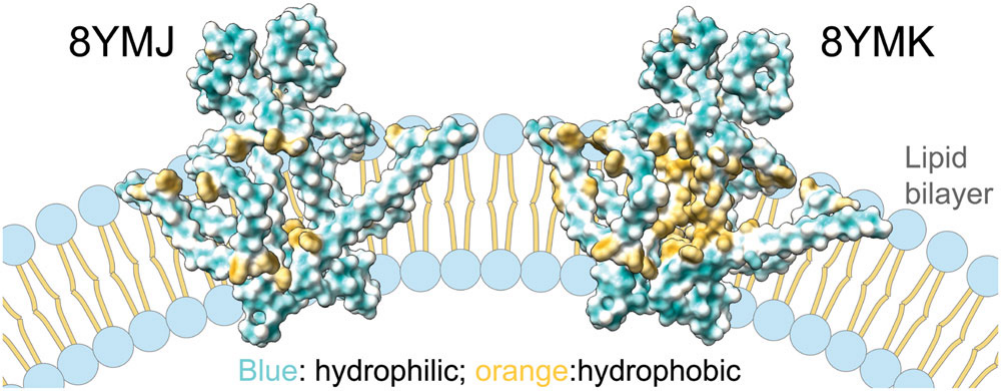
Proposed structural models of HBV SVP reveal predominantly hydrophilic protein surfaces. The S‐HBsAg structures, resolved by cryo‐EM (PDB code: 8YMJ and 8YMK), are shown in surface representation. Color coding: blue, hydrophilic regions; orange, hydrophobic regions.

We urge caution when interpreting HBV SVP lipid organization. All available evidence aligns more closely with a lipoprotein‐like structure, where lipids stabilize specific regions without forming a classical bilayer. This interpretation better reflects the biochemical and structural data and challenges the claim of a traditional lipid bilayer in SVPs.

## Conflicts of Interest

The authors declare no conflicts of interest.

## Data Availability

The authors have nothing to report. No new data were generated for this article. The atomic models shown in Figure 1 can be accessed from the Protein Data Bank (PDB) under accession codes 8YMJ and 8YMK.
